# HPLC-MS/MS Phenolic Characterization of Olive Pomace Extracts Obtained Using an Innovative Mechanical Approach

**DOI:** 10.3390/foods13020285

**Published:** 2024-01-16

**Authors:** Ilaria Grigoletto, Patricia García Salas, Enrico Valli, Alessandra Bendini, Federico Ferioli, Federica Pasini, Sebastián Sánchez Villasclaras, Roberto García-Ruiz, Tullia Gallina Toschi

**Affiliations:** 1 Department of Agricultural and Food Sciences, Alma Mater Studiorum—Università di Bologna, Piazza Gabriele Goidanich 60, 47521 Cesena, Italy; ilaria.grigoletto2@unibo.it (I.G.); patricia.garciasalas@unibo.it (P.G.S.); enrico.valli4@unibo.it (E.V.); federico.ferioli@unibo.it (F.F.); federica.pasini5@unibo.it (F.P.); 2 University Institute of Research on Olive Groves and Olive Oils, GEOLIT Science and Technology Park, University of Jaen, 236 Mengibar, Spain; ssanchez@ujaen.es (S.S.V.); rgarcia@ujaen.es (R.G.-R.); 3 Department of Agricultural and Food Sciences, Alma Mater Studiorum—Università di Bologna, Viale Fanin, 40, 40127 Bologna, Italy; tullia.gallinatoschi@unibo.it

**Keywords:** olive pomace, phenolic compounds, HPLC-MS/MS, by-product valorization

## Abstract

Olive pomace results from the production of olive oil. Even if olive pomace represents a potential environmental problem, it contains phenolic compounds, which are widely recognized for their beneficial properties for human health. In this study, an innovative and sustainable technological approach to extract phenolic compounds from fresh olive pomace, based on food-grade solvent instead of those usually adopted, is investigated. Characterization and shelf-life evaluation of the hydroalcoholic extracts obtained from the procedure developed for different industrial purposes were also carried out. The phenolic fractions of the different samples were studied with the Folin–Ciocâlteu method to quantify that the total reducing molecules and HPLC-MS/MS analysis was used to define the profile through the identification and quantification of 42 compounds, belonging to five chemical families. Regarding shelf-life, the hydroalcoholic extract showed no significant reduction in phenolic content, for both instrumental evaluations, retaining most of the phenolic compounds present in the raw material; negative attributes were not perceived by sensory evaluation. Thus, these lab-scale results can be the starting point to develop a procedure that is suitable for a real olive mill, representing a valorization strategy in a circular economy and the perspective of new business models.

## 1. Introduction

Nowadays, in the context of the global environmental and food crisis, it is becoming more and more important to reach a high standard of sustainability at different levels, particularly in terms of minimizing the environmental impact of by-products produced by agro-industries and the costs associated with their effective management [1]. The agri-food sector has been identified as having the most relevant environmental impact. For this reason, the European Commission declared that it is important to improve the sustainability of this sector to benefit the entire planet [2]. Due to the scarcity of resources and concerns of climate change, many governments are now trying to apply the principles of the circular economy in all sectors of the economy [2], including the agri-food system.

In this sense, researchers are paying more attention to waste and by-products to find solutions for their valorization, since they represent not only a potential source of energy but also bioactive molecules [3].

One of the central food systems in the Mediterranean Basin is focused on olive cultivation and olive oil production [4] and around 70% of the global production is concentrated mainly in Spain, Greece, Italy, and Portugal [5].

Olive oil production, as an agro-industrial activity, generates huge amounts of waste and by-products with an environmental footprint in short periods of time; olive pomace represents the main solid or semi-solid by-product obtained from mechanical extraction of olive oil from olive fruits [6,7]. As reported by Argun et al., 2023 [8], the annual olive oil production in the Mediterranean region is approximately 4 million tons, generating about 16 million tons of pomace waste. This product is essentially composed of skin, pulp, stone fragments, water, and oil, with a moisture content that varies from 50% to 65%, depending on the type of decanter used. The so-called two- and three-phase decanters, defined also as two- and three-outlet decanters since the separation does not actually lead to products in different phases, namely solid, liquid, and gas, are generally applied in olive oil production. In a two-phase system, a relevant amount of olive pomace with high moisture is obtained, while less wastewater is generated compared to a three-outlet decanter [8]. In contrast, in a three-outlet decanter, the product is separated into oil, water, and a solid material (stone fragments and olive pulp). The water-saving decanter, “ARA”, is an evolution of the three-outlet decanter, that, thanks to a special design based on a longer cylindrical part of the drum and shorter beach sections complemented by a pressure cone drum, allows the use of less water to dilute olive paste and produces an oil that is richer in phenolic compounds [9]. Unused pomace, if disposed of improperly, could have serious environmental consequences since it contains organic compounds with phytotoxic properties and a high demand for oxygen, which are potentially dangerous for soil and groundwater [10,11]. However, this material can be used for the extraction of residual oil [12] and, after suitable treatments, because of its organic content, as a soil amendment to improve soil properties leading to increased soil productivity and production of biogas. The pomace is sometimes used as animal feed or composted to produce a stable organic soil amendment with fertilization value. It is also used as a substrate for the production of activated carbon, as a source of bio-pesticides, in co-firing with coal at power stations, and as a source of residual oil for the soap industry [13]. In addition, olive pomace may be a natural and low-cost source of bioactive compounds with beneficial and healthy properties. Due to their hydrophilic nature, phenolic compounds are abundant in pomace, since only 2% are transferred to olive oil during oil extraction, whereas 98% remain in the by-product, including both simple phenols (e.g., hydroxytyrosol and tyrosol) and more complex molecules, generally named polyphenols [14,15]; for this reason, the term “phenolic compounds” is used herein, while in several frameworks, including the EU Reg. 432/2012 regarding olive oil health claims, they are called “polyphenols”.

Among these compounds, secoiridoids and simple derivatives (hydroxytyrosol and tyrosol) are some of the most valuable molecules in terms of antioxidant, antimicrobial, and health properties. For these reasons, they show potential in industrial applications, as antioxidants, fertilizers, and antibacterial drugs, as well as gelling and stabilizing agents in food products [14]. Therefore, the study of the olive pomace fraction for optimization of the recovery of high-value-added compounds and their commercial use is highly relevant [14,15]. Different approaches are present in the literature for the recovery of bioactive molecules from olive pomace: the most conventional methods use hydroalcoholic mixtures, while a new generation of greener and more environmentally friendly solvents, called deep eutectic solvents (DESs), is under investigation [12,16]. The emerging techniques adopted for the phenolic extraction from olive pomace are mainly based on the use of ultrasound (UAE) and pressurized liquid extraction (SFE) to prevent the degradation of thermolabile compounds. Other innovative techniques are microwave assisted extraction (MAE), pulsed electric field (PEF), high voltage electrical discharge (HVED), high pressure-high temperature (HPHT) [3,17], and membrane technologies (e.g., ultrafiltration (UF), nanofiltration (NF), or reverse osmosis (RO)) in which valuable antioxidant compounds can be kept in the retentate (RO, NF) or distributed between the permeate and the retentate streams (UF) [14]. For this reason, the application of a mechanical approach on fresh olive pomace, avoiding such conventional or emerging techniques, could be a more sustainable option to extract phenolic compounds from olive pomace.

In the European context, the PRIMA project SUSTAINOLIVE (Grant Agreement No. 1811) aims to improve the sustainability of the olive oil sector through the implementation and promotion of innovative and sustainable solution sets in management practices, including valorization of by-products. With this purpose, among the established activities, one was aimed at the innovative technological valorization of olive pomace by obtaining extracts that are rich in phenolic compounds and potentially usable in different industrial sectors, such as the pharmaceutical, food, and cosmetic sectors.

Several epidemiological studies indicate that dietary intake of foods rich in antioxidants can prevent diseases such as aging, cancer, cardiovascular disease, and diabetes. In a recent study, olive-derived polyphenols were reported to have particular pharmacological effects by several mechanisms, such as participating in the activation of different signaling pathways involved in the prevention of inflammation, oxidative stress, and insulin resistance [18]. In addition, phenolic compounds can be adopted in the food industry as “natural” food additives with antioxidant properties to extend the shelf-life of products, thus preventing nutritional losses and the formation of harmful substances such as free radicals. For these reasons, the use of extracts obtained from olive pomace rich in phenolic compounds can represent a low-cost and sustainable strategy [18].

In this framework, this study proposes a new lab-scale method for the extraction of phenolic compounds (i.e., by a mechanical approach and using food-grade solvents different from those usually adopted) by obtaining extracts rich in phenolic compounds, as well as their characterization and evaluation of shelf-life.

## 2. Materials and Methods

### 2.1. Samples

**Figure 1:**
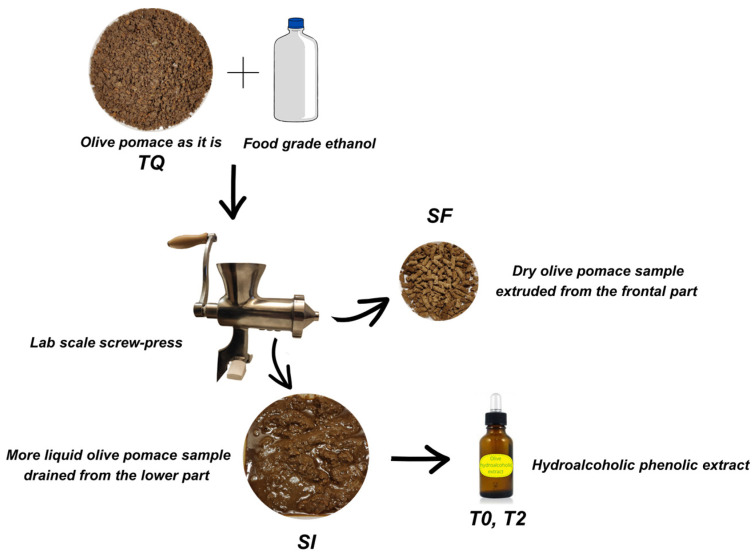
Flow chart representing the process to obtain the samples.

The samples were taken from the olive oil mill of the “Terra di Brisighella” agricultural cooperative (Brisighella, Ravenna, Italy) and produced during the 2020/21 campaign. The variety of the processed olives was ‘Nostrana di Brisighella’. The sampling was performed in the olive oil mill directly at the exit of the three-outlet decanter extractor “A.R.A.” (Water Saving Technology) after the application of a vibrating screen to remove water; samples were subsequently stored in a freezer at a temperature of −18 °C. A mechanical approach (using a lab scale screw-press) was applied on the olive pomace by adding a mixture of food grade ethanol and water (80:20%, *v*/*v*) to obtain two types of samples: one from liquid drained from the lower part of the screw press (named SI) and one extruded from the frontal part (named SF). The phenolic fractions were investigated using these samples, including olive pomace as it is (named TQ). The more liquid sample drained from the lower part of the screw-press (SI) was selected as the most suitable to obtain stable hydroalcoholic phenolic extracts (T0). The process and the definitions of the samples are reported in Figure 1. The procedure included the filtration of SI (by using a Buchner filter), the evaporation of the liquid fraction (using rotary evaporator at 38 °C), and the recovery of the residue with 50 mL of food grade ethanol.

### 2.2. Dry Matter Determination

Dry matter content was determined gravimetrically as the mass loss of 10 g of samples TQ, SI, and SF, at 105 ± 1 °C, until constant weight. The analysis was performed on three replicates. The results were expressed the amount of total and individual phenolics on a dry basis. The dry matter content, expressed as % (d.m.%), was calculated using the following formula: dry matter weight (g)/wet matter weight (g) ×100.

### 2.3. Determination of Yield (YF) and Technological Retention Factor (TRF)

YF was assessed for both SI and SF products obtained from olive oil pomace as it is (TQ) after the mechanical treatment described above. YF represents the amount in weight which is retained after technological treatment and was calculated on both a fresh and dry basis as the product weight-to-raw material weight ratio. The retention factor was named “TRF” (technological retention factor) to specify the correlation to the technological process. This was calculated for the total phenolic amount by multiplying the YF by the ratio between the phenolic amount determined in the technological processed olive pomace or hydroalcoholic extract and the phenolic amount in olive pomace as it is (TQ).

RF = (phenolic amount in processed olive pomace or pomace product)/(phenolic amount in olive pomace as it is, TQ) × YF.

### 2.4. Phenolic Extraction Procedure

Before the phenolic extraction, TQ, SI, and SF samples were freeze-dried and ground until the analysis for analytical reasons to optimize the characterization. For the extraction, 2 g of the freeze-dried grounded samples were weighed in a glass centrifuge tube. Next, 20 mL of the ethanol/water mixture (80:20%, *v*/*v*) was added. The preparation was shaken and an ultrasound bath (39 kHz constant frequency) was used at an ambient temperature for 15 min in order to favor the complete extraction. Subsequently, the solution was centrifuged for 15 min at 3500 rpm at room temperature. The polar phase was removed carefully with the aid of a pipette and the extraction process was repeated twice. Finally, the three extracts were combined, evaporated in a rotary evaporator at room temperature under vacuum until dryness, and the residue was dissolved in 2 mL of ethanol/water (1:1%, *v*/*v*).

### 2.5. Determination of the Total Reducing Molecules by the Folin–Ciocâlteu Method

Before spectrophotometric analyses, all extracts were properly diluted to obtain absorbance values in the range consistent with the Beer–Lambert law that relates absorbance to the chromophore concentration. In particular, TQ and SF samples were diluted 20-fold in ethanol/water (4:1%, *v*/*v*), whereas SI underwent a 40-fold dilution in the same mixture. T0 and T2 extracts were all diluted 5-fold in pure ethanol. The Folin–Ciocâlteu was performed according to Singleton and Rossi (1965) [19] with some modifications and can be briefly described as follows. First, 7.3 mL of water, 0.2 mL of each different diluted olive pomace extract, 0.5 mL of Folin–Ciocâlteu reagent, and 2.0 mL of 15% (*w*/*v*) sodium carbonate were transferred to a 10-mL PTFE screw cap glass tube and shaken for 5 s. The mixture was then kept in the dark at room temperature and after 2 h and not over 8 h, the absorbance of the solution was read at 750 nm in a single beam spectrophotometer (mod. UV-5600) from Hinotek (Ningbo, China). A gallic acid calibration curve was constructed to quantify reducing substances. From a stock solution (c = 2.01 mg mL^−1^) in methanol/water 4/1 (*v*/*v*), diluted solutions were prepared in the same solvent mixture in a concentration range of 0.0025–0.25 mg mL^−1^ (seven calibration points, r^2^ > 0.99). Observed absorbance values were corrected subtracting the absorbance of a blank sample prepared by replacing the hydroalcoholic extract with 0.2 mL of ethanol/water 4/1 (*v*/*v*) (TQ, SI, and SF samples) or ethanol (T0 and T2 ethanolic extracts). The procedure described above was carried out twice on each replicate of each extract and also on blanks and standard solutions.

### 2.6. Phenolic Identification and Quantification by High-Performance Liquid Chromatography–Tandem Mass Spectrometry (HPLC-MS/MS)

Phenolic compounds were identified and quantified using a liquid chromatography system HP 1290 Infinity Series equipped with a binary pump (mod. G4220B), a thermostatted column compartment (mod. G1316C), an autosampler (mod. G4226A), and an autosampler thermostat (mod. G1330B). The HPLC system was coupled with a triple quadrupole (QqQ) mass spectrometer (mod. G6420A). Both the HPLC system and mass spectrometer were from Agilent Technologies (Santa Clara, CA, USA). Experiments were carried out on different olive pomace fractions choosing multiple reaction monitoring (MRM) as scan type whereas ions were generated by an atmospheric pressure ionization-electrospray source (API-ES). In MRM mode, the first quadrupole (Q1) selects only specific precursor ions (generally molecular ions) to pass to the collision cell (second quadrupole, Q2) where the parent ions collide with N_2_ and undergo a further dissociation into product fragments. The third quadrupole (Q3) monitors and only lets product ions of a single *m*/*z* pass through to the detector. After preliminary studies, the following conditions were adopted throughout the trials: polarity, negative; drying gas (N_2_) temperature, 350 °C; nebulizer pressure, 50 psi; gas flow, 11 L min^−1^; capillary voltage, 3000 V; collision gas, nitrogen; and cell accelerator voltage, 7 V. Other parameters such as selected parent and product ions for each compound, fragmentor voltage, and collision energy used for each transition are specified in paragraph 3.2.2. Compound separation was obtained in a gradient mode on a Poroshell 120 SB-C18 (100 × 3.0 mm, 2.7 μm particle size) column from Agilent Technologies equipped with a guard cartridge Gemini NX (4.0 × 3 mm i.d.) from Phenomenex (Torrance, CA, USA). The solvent system was mobile phase A: 1% (*v*/*v*) acetic acid in water and mobile phase B: acetonitrile. Ethanol/water 4/1 (*v*/*v*) was used as a cleaning solution for the autosampler syringe needle before and after injection. All solvents were of chromatographic grade. The flow rate was 0.6 mL min^−1^ and the injection volume and column temperature were set at 2.5 μL and 35 °C, respectively. The gradient program was as follows: 0–12.5 min, 95 to 70% A; 12.5–17.5 min, 70 to 40% A, 17.5–20.0 min, 40 to 5% A; 20–26 min, 5% A, 26–30 min, 5 to 95% A; post run time: 10 min; and total method time: 40 min. Mobile phase A and syringe needle cleaning solution were preliminarily filtered on a nylon membrane filter (diameter: 47 mm; pore dimension: 0.45 μm). All mobile phases and cleaning solution were degassed by ultrasonic bath before HPLC analyses. Data were processed by the software MassHunter Workstation Software-Qualitative analysis ver. B.06.00 from Agilent Technologies. On the basis of previous investigations [20,21,22] dealing with the characterization of the olive pomace phenolic profile by HPLC-MS, we selected the main phenolic compounds that were identified in the aforementioned studies and the fragments that were generated and detected from the fragmentation of the molecular ion of each compound. Next, a series of MRM experiments were performed including all the possible transitions of each molecular ion to the selected fragments. These trails were carried out under different experimental conditions, combining three values of fragmentor voltage (60, 100, and 150 V) and three values of collision energy (10, 20, and 30 eV) in order to identify the best transition, fragmentor, and collision energy values for each compound that give rise to the most abundant peak in the MRM mode and to be successively used for quantification in the optimized MRM method. Other minor transitions related to the same parent ion were used to confirm a compound’s identity. Phenolic quantification was performed by constructing calibration curves of standard compounds representing different chemical classes. Details of each calibration curve are illustrated in paragraph 3.2.2.

### 2.7. Shelf-Life Study of the Hydroalcoholic Extract

On the hydroalcoholic extract, phenolic compounds were characterized and stability during a shelf-life study was assessed, including both sensory (descriptive analysis) and compositional (Folin–Ciocâlteu method, UHPLC-DAD, HPLC-MS/MS) evaluations. In particular, the shelf-life study was performed for two months [samples were coded as T0 (time zero) and T2 (after two months of storage)] and carried out on the selected extract stored at room temperature and dark conditions. In addition, a descriptive analysis was carried out by a panel composed of 8 panelists, trained for virgin olive oil sensory assessment, of the University of Bologna Professional Committee, through olfactory evaluation. Panelists were asked to exclude the perception of ethanol (a reference of food grade ethanol, in a vial, was given to them for this purpose). The assessors (8–12 per session) were all volunteers who were aware and informed about procedures and risks. The panelists were previously informed that they should evaluate only by smelling the hydroalcoholic extract obtained using food grade ethanol.

### 2.8. Data Analysis

In these experiments, significant differences (*p* < 0.05) among means were determined by one-way analysis of variance (ANOVA) with Tukey’s post-hoc test and two-sample *t*-test, using XLSTAT 2023.1.1 software (Addinsoft, New York, NY, USA).

## 3. Results and Discussion

### 3.1. Moisture and Mass Yields

The wet and dry matter of SI and SF from the TQ, SI, and SF are reported in Table 1. The TQ, namely olive pomace as it is, result (53.07% of dry matter) is in line with those found in the literature, in which olive pomace derived from a three-outlet decanter extractor “A.R.A.” was reported by Sicari et al., 2023, to have a moisture content of 55% [9].

Therefore, on average, 72% and 50% of the wet and dry matter, respectively, of the olive pomace was obtained in the SI fraction. The yield on wet (16%) and dry (18%) basis of the SF was similar.

### 3.2. Characterization of Olive Pomace Fractions

#### 3.2.1. Total Reducing Molecules of Olive Pomace Fractions

The content of reducing compounds was the highest for the SI fraction (Table 2), which was 3.5- and 2.18-fold higher than that of the SF fraction and the original olive pomace values for the latter were significantly higher than those of the SF fractions. This is probably due to the mechanical action of the screw-press and the use of the mixture of ethanol and water that favor the extraction of phenolic compounds. SI was selected as the most suitable sample to obtain the phenolic hydroalcoholic extract since a higher content of reducing compounds was obtained.

The results related to TQ, i.e., olive pomace as it is, are in line with those found in the literature, as reported by Tapia-Quirós et al. (2020) [23], in which different samples of olive pomace showed a range of 2050–8050 mg gallic acid kg olive pomace^−1^.

#### 3.2.2. Identification and Quantification of the Phenolic by High-Performance Liquid Chromatography–Tandem Mass Spectrometry (HPLC-MS/MS)

More than 40 compounds were identified. For each of the main phenolic compounds previously determined in olive pomace extracts, a number of potential product ions that could be originated from the fragmentation of the corresponding molecular ion were selected. Once the best transition was chosen, parameters such as fragmentor voltage and collision energy were also optimized. The results are shown in Table 3.

A total of 42 phenolic compounds were tentatively identified as summarized in Table 3, including the retention time, experimental *m*/*z*, and fragments. The compounds identified belong to different chemical families, such as hydroxycinnamic acids, organic acids, simple phenols, flavonoids, and iridoids. The phenolic compounds were identified taking the sample TQ as reference. In both SI and SF samples, all 42 compounds were found. Below, the identification of the phenolic compounds is described based on their chemical class.

For hydroxycinnamic acids, verbascoside and its isomers (ph28, ph3, ph7) were identified at retention times of 7.98, 1.18, and 2.18 min, respectively. Verbascoside presents a strong fragment at *m*/*z* 161 and another fragment at *m*/*z* 461, according to the literature [22,24]. Hydroxyverbascoside, with a retention time of 6.14 min and *m*/*z* of 621, showed other fragments at *m*/*z* 529 and 459. In 2012, it was also previously reported by Cardinali et al. in olive mill wastewater [25]. At a retention time of 2.39 min and [M-1] of 355, *p*-Cumaroyl-aldarate (ph10) was identified by comparison of mass spectra found in the literature and shows the strongest fragment at *m*/*z* 209. The other fragments are reported in Table 3. Compound 17 (ph17) was identified as caffeic acid at a retention time of 4.36 min. Other derivates of caffeic acid were found at retention times of 3.62 and 9.20 min and were identified as caffeoyl-hexoside (ph12) and caffeoyl-6-secologanoside (ph34), respectively. The fragment pattern, according to the literature, is *m*/*z* 135 for ph17, ph12, and *m*/*z* 161 for the ph34 [21,22]. Chlorogenic acid was identified at retention time 3.77 min, showing a fragment at 191, characteristic of this compound, as described by Malapert et al., 2018 [22]. *p*-coumaric acid (ph22) and one derivate, *p*-coumaroyl-hexoside (ph14), were found at 6.11 and 3.86 min, respectively. They both showed a strong fragment at 119, as reported by Malapert et al., 2018 [22]. At a retention time of 10.40, ph38 was identified as comselogoside, having a strong fragment 145 and other fragments at *m*/*z* 491, 389, and 345, as reported in Table 3 and by Rubio-Senent et al., 2013, and Romero et al., 2002 [24,26].

Other important phenolic compounds belong to the organic acids. The first compound identified belonging to this chemical family was quinic acid at 0.74 min, characterized by a strong fragment at *m*/*z* 127 and another at *m*/*z* 173, as reported by D’Antuono et al., 2014 [27]. Vallinic acid hexoside was found at 2.05 min, showing a strong fragment at *m*/*z* 167 with other fragments at 123 and 108 [21]. Finally, 4-hydroxyphenylacetic acid was identified at 5.74 min with the same fragmentation pattern reported by Obied et al., 2007 [21].

Regarding simple phenols, hydroxytyrosol (ph9), phenylethyl alcohol, was identified at a retention time of 2.32 min, with a fragmentation pattern characterized by strong fragments at *m*/*z* 123 and 153. Its derivates, such as hydroxytyrosol glucoside 1 and 2, were also identified, according to the information reported in the literature [22,27]. Dihydroxyphenylglycol was also detected at 1.06 min, with the same major fragment at *m*/*z* 169, according to the literature [21].

Flavonoids were represented by rutin (ph25), found at 7.39 min with a fragmentation pattern at *m*/*z* 609 and 301, as reported by Obied et al., 2007 [21]. Apigenin (ph42) was identified at a retention time of 13.76 and with characteristic fragments at *m*/*z* 227 and 161 [21]. Finally, luteolin was detected at 11.84 min, showing a strong fragment at *m*/*z* 133 and another fragment at *m*/*z* 151, as reported by Obied et al. in 2007 [21]. In addition, luteolin-O-rutinoside isomers 1, 2, and 3 and luteolin-glucoside isomers 1, 2, and 3 were also identified [21] (Table 3).

Regarding the iridoids family, the first phenolic compound identified was the hydroxylated DCMEA derivative (ph4) at retention time 1.86 min, with a strong fragment at *m*/*z* 199 and other fragments at 111 and 155 [28]. 1-beta-glucosyl-acyclodihydroelenolic acid (ph11) was identified at a retention time of 2.55 min, showing a precursor ion at *m*/*z* 407 and fragments at 313, 389, 375, 357, and 161. These compounds were derived from elenolic acid, found at 8.43 min, with a majority fragment at *m*/*z* 139. The fragmentation patterns of all these compounds coincide with those found by Malpert et al. [22]. Another relevant compound identified in our samples was oleuropein along with its derivates. Oleuropein aglycone was found at a retention time of 5.22 min, showing a strong compound at *m*/*z* 197 and another fragment at 153 [24,27]. Other compounds, 3,4-DHPEA-DEDA (oleuropein aglycone decarboxymethyl dialdehyde form), 10-hydroxy-DCMO aglycone, and oleuroside were identified at 4.00, 9.91, and 10.22 min, respectively. These compounds were identified by other authors in olive pomace [21,24]. Ligstroside aglycone was detected at 11.25 min, with a fragment at *m*/*z* 137 [21]. Minor compounds were found at 10.30 and 8.89 min and identified as 4-HPEA-DEDA (ligstroside aglycone decarboxymethyl dialdehyde form) and ligstroside derivative, respectively. Other compounds belonging to this chemical family identified in our olive pomace samples were oleoside, oleoside deoxyriboside, loganic acid glucoside, and nüzhenide. Retention times and fragmentation patterns are in Table 3, as previously described by other authors in olive pomace.

In order to quantify the most relevant compounds, calibration curves were prepared using the following standards: verbascoside, rutin, oleuropein, luteolin-7-glucoside, luteolin, apigenin, quinic acid, caffeic acid, and hydroxytyrosol in the range of 0.1–100 µg. In total, 10 calibration points were used for each, except for the oleuropein, in which 13 points were used (Table 4). All calibration curves showed good linearity (r^2^ > 0.99) between concentrations depending on the analyte studied. Since the amount of oleuropein aglycone (ph21) and elenolic acid (ph35) was much higher compared to other iridoids, in particular in samples TQ and SI, a second oleuropein calibration curve was constructed using higher concentrations of this standard compound. Similarly, a second calibration curve was built for caffeic acid to better quantify comselogoside (ph12), a caffeic acid derivative that showed a higher concentration compared to other compounds of the same chemical class.

The calibration curve of caffeic acid was used for the quantification of dihydroxyphenylglycol and hydroxycinnamic and organic acids, except for verbascoside and derivates and quinic acid, in which verbascoside and quinic acid calibration curves were used, respectively. The hydroxytyrosol curve was used for the quantification of phenylethyl alcohols (hydroxytyrosol, hydroxytyrosol glucoside isomer 1, hydroxytyrosol glucoside isomer 2). For flavonoids, specific calibration curves were used to quantify rutin, apigenin, and luteolin. Luteolin derivates were quantified with luteolin-7-glucoside. Lastly, iridoids and derivates were quantified using the oleuropein calibration curve.

Among the 42 compounds identified in TQ, as reported in Table 5, those that showed the highest concentration (between 8.322 to 866.60 mg kg^−1^ dry matter) were elenolic acid, oleuropein aglycone, quinic acid, hydroxylated DCMEA derivative, 1-beta-glucosyl-acyclodihydroelenolic acid, verbascoside, luteolin-glucoside isomer 1, rutin, ligstroside aglycone, and luteolin-glucoside isomer 3.

The total concentration was the highest for SI (2953.83 mg kg^−1^), followed by TQ (2007.34 mg kg^−1^) and SF (884.76 mg kg^−1^), which agrees with the results in Table 2. The results obtained by the total content in terms of phenolic compounds identified by HPLC-MS/MS are in line with those reported by Cea Pavez et al. [29], in which the total phenolic compounds were quantified in a range of 241 to 1141 mg kg^−1^ d.m, using different extraction approaches.

On the other hand, the results obtained in this present study are significantly different from data reported in previous investigations on the phenolic composition of olive pomace. For example, Malapert et al. [22] reported that hydroxytyrosol, two hydroxytyrosol glucosides, and tyrosol were the main phenolics in a French two-phase olive pomace sample, representing 43%, 19% (glucoside 1), 10% (glucoside 2), and 17% of the major phenolic compounds. Cardoso et al., 2005 [20], quantified different phenolic compounds in methanolic extracts recovered from olive pulp and olive pomace collected in Portugal by reverse phase HPLC. Even in this latter study, glucosides were the most abundant compounds and hydroxytyrosol-1′-β-glucoside and 6′-β-rhamnopyranosyl-oleoside both accounted for 25% of phenolic compounds; other compounds present at significant levels were 6′-β-glucopyranosyl-oleoside, oleoside, and luteolin-7-glucoside (co-eluting with verbascoside), amounting to 19%, 14%, and 8% of compounds.

**Figure 2:**
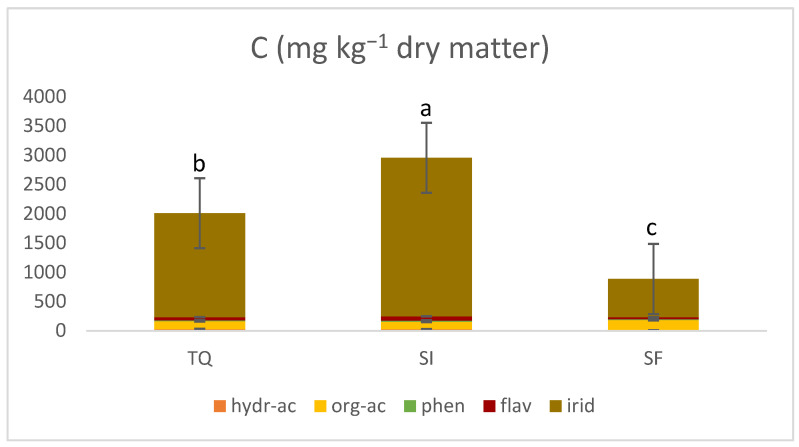
Total amount of 5 classes of phenolic compounds (“hydr-ac” for hydroxycinnamic acids, “org-ac” for organic acids, “phen” for simple phenols, “flav” for flavonoids and “irid” for iridoids class) in olive pomace as it is (TQ) and pomace derivatives (SI and SF). Different letters stand for significant differences in the total amount among TQ, SI, and SF.

Compounds belonging to the iridoids and derivates were the most abundant in each sample (TQ, SI, or SF), followed by the organic acid class (Figure 2).

### 3.3. Characterization and Shelf-Life of the Hydroalcoholic Extract

#### 3.3.1. Total Reducing Molecules

After two months, there was a slight reduction in the total reducing compounds. On average, the reduction was 5.1% of the initial total reducing compounds or a reduction as low as 0.54 mg per day during the two months.

#### 3.3.2. Phenolic Quantification by High-Performance Liquid Chromatography–Tandem Mass Spectrometry (HPLC-MS/MS)

**Figure 3:**
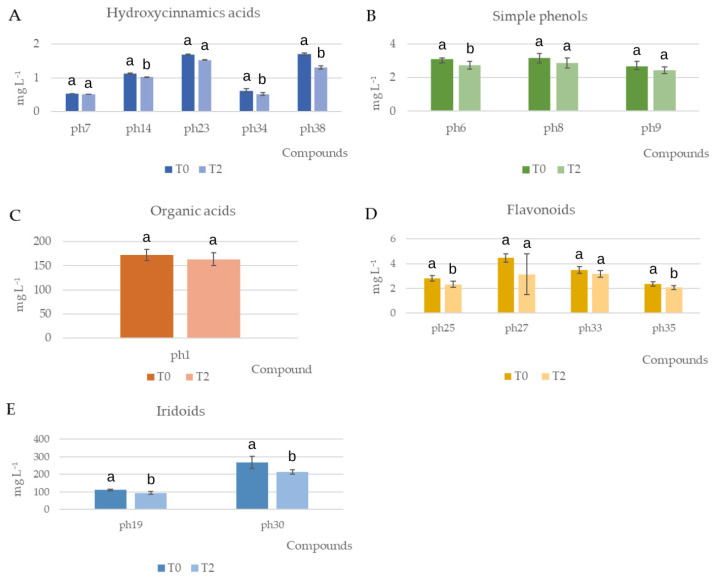
Concentration of the main compounds of hydroxycinnamic acids (**A**), simple phenols (**B**), organic acids (**C**), flavonoids (**D**), and iridoids (**E**) classes at T0 and T2. Different letters for a compound within a class stand for significant differences in the concentration in T0 and T2.

Altogether, 37 phenolic compounds (Table 3), of a total of 42, were identified in hydroalcoholic extracts (T0 and T2), belonging to the different chemical families previously found and identified in TQ. Consequently, the majority of the phenols present in the raw material (TQ) were also in the final extract. Even if the total amount of phenolic compounds, expressed as mg mL^−1^ of extract, in T0 and T2 (627.37 ± 49.21 and 537.55 ± 34.00, respectively) was significantly different (two-sample *t*-test, *p* < 0.05), a mean decrease of 5–15% can be considered acceptable. The decrease by class of phenolic compounds is shown in Figure 3.

The main compounds found in T0 and T2 for the hydroxycinnamic acids class were verbascoside-rha isomer 2 (ph7), *p*-coumaroyl hexoside (ph14), hydroxyverbascoside (ph23), caffeoyl-6-secologanoside (ph34), and comselogoside (ph38) (Figure 3). Traditionally, plants with high concentrations of verbascoside have been used in folk medicine to treat inflammation and microbial infections. Studies regarding anti-microbial and anti-fungal activities have been carried out but in general are purely observational and there are many mechanistic approaches [30]. In addition, comselogoside (ph38) showed antioxidant and radical scavenging activity [31]. Among these phenolic compounds, caffeic acid and derivates, such as caffeoyl-6-secologanoside (ph34), as major representatives exhibiting different health benefits such as antioxidant properties, may help prevent inflammation, cancer, neurodegenerative diseases, and diabetes [32].

Quinic acid (ph1) was the main phenolic compound quantified in the organic acids class, which were the second most abundant class (Figure 3). Quinic acid did not decrease in concentration between T0 and T2. Quinic acid is an organic acid that is present in several medicinal plants and has been studied for its biological properties. This compound appears to have antibacterial antioxidant, antiviral, antidiabetic, anticancer, anti-nociceptive, and analgesic properties, as well as anti-aging and protective effects [33].

Regarding simple phenols, we identified hydroxytyrosol (ph9) and relative glucosides isomer 1 and 2 (ph6 and ph9, respectively). Only ph6 showed a significant reduction at T2 of 12% of the initial value. Hydroxytyrosol (IUPAC name: 4-(2-hydroxyethyl)-1,2-benzenediol) derives from hydrolysis of oleuropein during maturation of olives and is known for its antioxidant activity. In addition, it may have anti-inflammatory, anti-atherogenic, and anti-thrombotic properties. In vitro studies have shown that, in addition to its antioxidant activity, it can improve endothelial dysfunction, lipid and hemostatic profiles, and has anti-inflammatory properties. Therefore, it may be considered as a neuroprotective, cardioprotective, chemopreventive, and anti-cancer agent [34]. Hydroxytyrosol-glucoside has been reported to be one of the most abundant compounds in olive pomace by Zhao et al. in 2022 [16].

The main compounds belonging to the flavonoids are shown in Figure 3. Among these, rutin (ph25) is one of the most significant flavonoids and is known to be an active radical scavenger. In particular, several olive leaf flavonoids such as rutin, quercetin, luteolin, and its glucosides are reported to be efficient radical scavengers, with some presenting superiority in offline determinations compared to oleuropein and/or hydroxytyrosol [35]. Luteolin-glucoside isomers 1 (ph27), 2 (ph33), and 3 (ph35) are reported to derive from luteolin (Figure 2), a compound that has been shown to have anti-inflammatory and antioxidant properties, which makes it capable of preventing cellular damage by scavenging the compounds containing reactive nitrogen and oxygen species [36]. Only two (ph25 and ph35) of the four flavonoids showed a reduction in concentration at T2 but were lower than 20%.

The most abundant compounds of the iridoid class, found at both T0 and T2, were oleuropein aglycone (ph19) and elenolic acid (ph30). Oleuropein is known to be the main phenolic molecule of extra virgin olive oil (EVOO). It is derived from the deglycosylation of oleuropein that is present in the leaves and stone fruits of *Olea europaea* during the maturation period and obtained by squeezing. Oleuropein aglycone is gaining increasing attention due to its favorable biological properties in Alzheimer’s disease and breast cancer and due to its anti-inflammatory, anti-hyperglycemic, anti-oxidative, and lipid-lowering properties [37]. Finally, elenolic acid (ph30) is a relevant phenolic compound since it is hypothesized that the antimicrobial effect of oleuropein may be due to its components hydroxytyrosol [26] and EA [38]. The reduction in concentration of both (ph19 and ph30) over two months was 15% and 20%, respectively.

#### 3.3.3. Sensory Analysis

The sensory evaluation was carried out by eight trained tasters of a virgin olive oil panel, through olfactory evaluation, and were asked to exclude the perception of ethanol and to evaluate other relevant defects or negative attributes for virgin olive oils (e.g., fusty-muddy sediment, musty-humid-earthy, winey-vinegary, rancid, and frostbitten olives) or positive notes. Negative attributes were not perceived by the tasters, while the definitions collected related mostly to olfactory notes resembling vanilla, caramel, red fruits, and olive fruits. The results are specified in Table 6.

These results highlighted that there is no degradation of the hydroalcoholic extract from a sensory point of view when the extraction process (extrusion and extraction) was carried out on freshly collected pomace. However, further studies need to be carried out by considering a period longer than 2 months.

### 3.4. Technological Retention Factor

**Figure 4:**
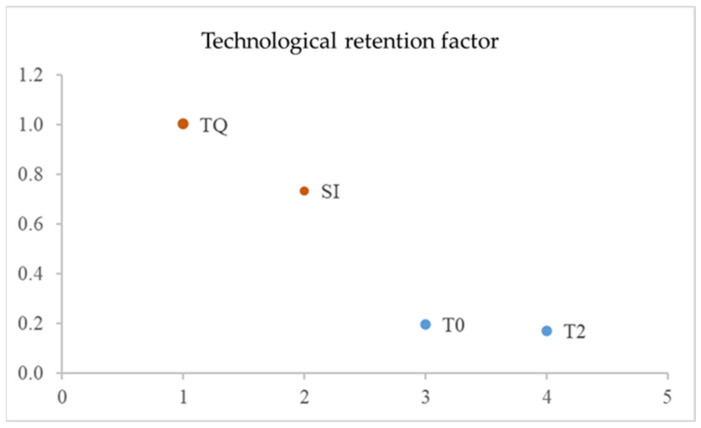
Technological retention factors (TRF, %), i.e., amount of phenolic compounds remaining in SI, T0, and T2 with respect to TQ.

From the determination of TRF, calculated as a percentage of TQ, 73% of the total phenolic compounds remain in SI and 17–20% in the hydroalcoholic extract at T0 (20%) and T2 (17%), respectively (Figure 4). This shows that not all the phenolic compounds present in TQ are recovered through the extraction procedure described herein. Further studies should focus on the improvement in the extraction conditions and shelf-life, in order to obtain phenolic hydroalcoholic extracts that are even richer in phenolic compounds and more stable.

## 4. Conclusions

This investigation contributes to the valorization of olive oil pomace, which is well-known for its high content of phenolic compounds; in fact, the extracts obtained herein are potentially usable in different industrial sectors, such as the pharmaceutical, food, and cosmetic sectors. The phenolic fraction of the raw material (olive pomace as it is, TQ), intermediate samples obtained by mechanical process (SI and SF), and the final product (hydroalcoholic extract, assessed at T0 and T2) were characterized. From the HPLC-MS-MS analysis, 42 phenolic compounds were identified and quantified. Next, the hydroalcoholic extract obtained, after 2 months of storage at room temperature, demonstrates stability over time: a reduction in the total reducing molecules and the different classes of phenolic compounds, was not observed, nor were sensory defects perceived by trained tasters. Thus, the developed method herein to obtain a phenolic extract is based on a sustainable mechanical approach and the use of food-grade ethanol, with a lower environmental impact overall compared to conventional extractive procedures, which often require organic solvents, potentially more toxic than ethanol, and massive energy consumption. The lab-scale results presented herein can be the starting point to set up a procedure to be implemented in a real olive mill. In such an industrial framework, it will be essential to make the process continuous by integrating the squeezing and extrusion of the fresh pomace in the traditional olive milling procedure.

While the present results are promising, future studies will be needed to render the procedure even more sustainable (e.g., more effectively concentrating/removing water in extracts) and assess the stability of extracts for a longer period of time.

## Figures and Tables

**Table 1:** Dry matter as % (d.m.%), wet and dry matter (g) and yields (TQ/TQ, SI/TQ, and SF/TQ) of TQ, SI, and SF.

Sample	Dry Matter (%)	Wet Matter (w.m,g)	Wet Matter Yield	Dry Matter (d.m.,g)	Dry Matter Yield
TQ	53.07	150.00	1.00	79.60	1.00
SI	36.53	108.32	0.72	39.57	0.50
SF	59.03	24.61	0.16	14.53	0.18

**Table 2:** Total content of reducing molecules, assessed by the Folin–Ciocâlteu method, on olive pomace samples as it is (TQ) and the SI and SF extruded fractions. Different letters stand for significant differences (ANOVA, Tukey’s test; *p* < 0.05).

Sample	Reducing Substance Content (mg Gallic Acid kg Olive Pomace^−1^ d.m.)	SD
TQ	6750.6 ^b^	1126.5
SI	14741.5 ^a^	1356.5
SF	4164.2 ^c^	96.15

**Table 3:** Common names, tag, chemical family (organic acids, simple phenols, hydroxycinnamic acids, iridoids, and flavonoids), retention times (RTs), precursor and product ions chosen for MRM experiments, fragmentor voltage, collision energy (CE), and other transitions of the phenolic compounds identified in SI (more liquid sample) and SF (extruded sample), and the hydroalcoholic extract (T0, T2).

Compound Name	Tag	Chem.Family	RT (min)	Precurson Ion [M-1]	Product Ion	Fragmentor Voltage	CE	Other Transitions	SI	SF	T0	T2
Quinic acid	ph1	org-ac	0.74	191	127	150	20	173	x	x	x	x
Dihydroxyphenylglycol	ph2	phen	1.06	169	151	60	10		x	x	x	x
Verbascoside-rha isomer 1	ph3	hydr-ac	1.18	477	161	100	10		x	x		
Hydroxylated DCMEA derivative	ph4	irid	1.86	199	111	60	10	155	x	x	x	x
Vanillic acid hexoside	ph5	org-ac	2.05	329	167	150	10	123, 108	x	x	x	x
Hydroxytyrosol glucoside isomer 1	ph6	phen	2.11	315	153	150	10	123	x	x	x	x
Verbascoside-rha isomer 2	ph7	hydr-ac	2.18	477	161	100	10		x	x	x	x
Hydroxytyrosol glucoside isomer 2	ph8	phen	2.25	315	153	150	10	123	x	x	x	x
Hydroxytyrosol	ph9	phen	2.32	153	123	100	10		x	x	x	x
p-Coumaroyl aldarate	ph10	hydr-ac	2.39	355	209	100	10	191, 147, 129	x	x		
1-Beta-glucosyl-acyclodihydroelenolic acid	ph11	irid	2.55	407	313	150	10	389, 375, 357, 161	x	x	x	x
Caffeoyl-hexoside	ph12	hydr-ac	3.62	341	179	150	10	135	x	x	x	x
Chlorogenic acid	ph13	hydr-ac	3.77	353	191	100	10		x	x	x	x
p-Cumaroyl-hexoside	ph14	hydr-ac	3.86	325	119	100	30	163	x	x	x	x
Oleoside	ph15	irid	3.90	389	345	150	10	209	x	x	x	x
3,4-DHPEA-DEDA	ph16	irid	4.00	319	195	100	30		x	x		
Caffeic acid	ph17	hydr-ac	4.36	179	135	100	10		x	x	x	x
Oleoside deoxyriboside	ph18	irid	4.91	505	389	150	10	345, 121	x	x	x	x
Oleuropein aglycone	ph19	irid	5.22	377	197	100	10	153	x	x	x	x
Loganic acid glucoside	ph20	irid	5.36	537	375	60	10	179	x	x	x	x
4-Hydroxyphenylacetic acid	ph21	org-ac	5.74	151	108	60	20		x	x	x	x
p-Coumaric acid	ph22	hydr-ac	6.11	163	119	100	10		x	x	x	x
Hydroxyverbascoside	ph23	hydr-ac	6.14	639	621	150	20	529, 459	x	x	x	x
4-HPEA-DEDA	ph24	irid	7.30	303	285	100	10	179	x	x		
Rutin	ph25	flav	7.39	609	301	100	30	179	x	x	x	x
Luteolin-O-rutinoside isomer 1	ph26	flav	7.59	593	285	150	30		x	x	x	x
Luteolin-glucoside isomer 1	ph27	flav	7.83	447	285	150	20		x	x	x	x
Verbascoside	ph28	hydr-ac	7.98	623	161	100	30	461	x	x	x	x
Luteolin-O-rutinoside isomer 2	ph29	flav	7.98	593	285	150	30	447	x	x	x	x
Elenolic acid	ph30	irid	8.43	241	139	60	10	165, 127, 121, 101	x	x	x	x
Luteolin-O-rutinoside isomer 3	ph31	flav	8.46	593	285	150	30		x	x	x	x
Nüzhenide	ph32	irid	8.62	685	523	100	10	453, 421, 403, 299	x	x	x	x
Luteolin-glucoside isomer 2	ph33	flav	9.10	447	285	150	20		x	x	x	x
Caffeoyl-6-secologanoside	ph34	hydr-ac	9.20	551	161	100	30	507, 389, 341, 281, 252, 221, 179	x	x	x	x
Luteolin-glucoside isomer 3	ph35	flav	9.81	447	285	150	20		x	x	x	x
10-Hydroxy-DCMO aglycone	ph36	irid	9.91	335	199	100	10	155, 111	x	x	x	
Oleuroside	ph37	irid	10.22	539	275	150	20	469, 377, 307	x	x	x	x
Comselogoside	ph38	hydr-ac	10.40	535	145	150	20	491, 389, 345, 307, 265, 163	x	x	x	x
Ligstroside derivative	ph39	irid	10.89	655	291	150	20	361, 259	x	x	x	x
Ligstroside aglicone	ph40	irid	11.25	361	137	100	10		x	x		
Luteolin	ph41	flav	11.84	285	133	150	30	151	x	x	x	x
Apigenin	ph42	flav	13.76	269	227	150	20	161	x	x	x	x

Org-ac (organic acids); Phen (simple phenols); Hydr-ac (hydroxycinnamic acids); Irid (iridoids); Flav (flavonoids).

**Table 4:** Main parameters of the calibration curves were built to quantify phenolic compounds identified in olive pomace as it is (TQ) and pomace derivatives (SI and SF).

Analyte	LOD (mg L^−1^)	LOQ (mg L^−1^)	Concentration Range (mg L^−1^)
Verbascoside	0.01	0.04	0.10–22.30
Rutin	0.01	0.04	0.10–24.80
Oleuropein ^A^	0.01	0.03	0.10–99.00
			99.00–831.90
Luteolin-7-glucoside	0.003	0.01	0.10–24.60
Luteolin	0.001	0.002	0.10–24.60
Apigenin	0.01	0.02	0.10–4.80
Quinic acid	0.02	0.08	0.25–101.00
Caffeic acid ^B^	0.02	0.07	0.10–5.00
			0.10–25.20
Hydroxytyrosol	0.02	0.06	0.10–24.70

^A^ The first calibration curve was used to quantify iridoids, whereas the second one for the quantification of ph1 in samples TQ and SI and ph35 in all samples; ^B^ The first calibration curve was used to quantify caffeic acid and other caffeic acid derivatives, whereas the second one was only for the quantification of compound ph12 in all samples.

**Table 5:** Common names, tag (phenolic compound codification), average concentration, and standard deviation (SD) of compounds identified in olive pomace as it is (TQ), pomace products obtained after mechanical treatment (SI and SF), and hydroalcoholic extracts (T0 and T2).

		Samples Average Concentrations									
Compound Name	Tag	TQ (mg/kg d.m.) ^A^	SD	SI (mg/kg d.m.)	SD	SF (mg/kg d.m.)	SD	T0 (mg/L)	SD	T2 (mg/L)	SD
Quinic acid	ph1	130.78	9.62	121.92	5.64	172.16	5.4	172.54	11.74	163.71	12.81
Dihydroxyphenylglycol	ph2	0.14	0.02	0.26	0.03	0.31	0.06	0.71	0.07	0.61	0.09
Verbascoside-rha isomer 1 ^B^	ph3	0.65	0.15	0.38	0.05	0.07	0.04	ND	-	ND	-
Hydroxylated DCMEA derivative ^C^	ph4	81.55	15.39	84.78	5.87	34.41	2.16	20.99	2.71	19.6	2.94
Vanillic acid hexoside	ph5	2.62	0.16	2.51	0.14	1.61	0.06	1.41	0.04	1.31	0.12
Hydroxytyrosol glucoside 1	ph6	2.78	0.40	6.84	0.35	4.5	0.1	3.09	0.07	2.73	0.23
Verbascoside-rha isomer 2 ^B^	ph7	0.24	0.13	1.04	0.11	0.12	0.04	0.54	0.09	0.51	0.10
Hydroxytyrosol glucoside 2	ph8	7.57	0.93	11	0.48	5.31	0.20	3.17	0.25	2.88	0.30
Hydroxytyrosol	ph9	4.25	0.37	4.39	0.43	3.04	0.20	2.68	0.29	2.43	0.20
p-Coumaroyl aldarate	ph10	0.29	0.07	0.47	0.08	0.09	0.02	ND	-	ND	-
1-beta-Glucosyl-acyclodihydroelenolic acid	ph11	33.75	3.02	37.04	1.26	15.03	1.73	13.21	0.23	11.58	1.83
Caffeoyl-hexoside	ph12	0.12	0.01	0.24	0.02	0.11	0.02	0.08	0.01	0.07	0.01
Chlorogenic acid	ph13	0.29	0.04	0.3	0.04	0.06	0.01	0.04	0.02	0.02	0
p-Cumaroyl-hexoside	ph14	2.33	0.18	2.76	0.08	1.26	0.04	1.12	0.06	1.02	0.08
Oleoside	ph15	2.89	0.64	3.63	0.63	0.68	0.21	0.6	0.18	0.5	0.17
3,4-DHPEA-DEDA ^D^	ph16	TR	-	TR	-	1.66	0.26	ND	-	ND	-
Caffeic acid	ph17	0.22	0.05	0.46	0.03	0.13	0.02	0.09	0.01	0.07	0.01
Oleoside deoxyriboside	ph18	3.00	0.77	3.76	0.56	0.51	0.14	TR	-	TR	-
Oleuropein aglycone	ph19	765.72	223.78	1224.53	153.93	118.97	9.22	111.95	4.53	94.76	8.34
Loganic acid glucoside	ph20	2.30	0.31	4.41	0.24	1.71	0.18	1.57	0.18	1.25	0.19
4-Hydroxyphenylacetic acid	ph21	0.26	0.03	0.34	0.04	0.92	0.05	0.05	0.01	0.05	0.01
p-Coumaric acid	ph22	0.42	0.04	0.4	0.01	0.56	0.01	0.05	0.01	0.03	0.01
Hydroxyverbascoside	ph23	3.86	0.29	4.1	0.53	1.02	0.24	1.69	0.15	1.53	0.23
4-HPEA-DEDA ^E^	ph24	0.47	0.22	1.18	0.15	2.16	0.18	ND	-	ND	-
Rutin	ph25	9.14	1.59	14.8	1.81	3.82	0.27	2.81	0.22	2.32	0.24
Luteolin-O-rutinoside isomer 1	ph26	2.13	0.21	4.03	0.32	0.99	0.07	0.92	0.08	0.74	0.06
Luteolin-glucoside isomer 1	ph27	12.02	1.05	19.99	1.57	9.51	0.69	4.47	0.34	3.15	1.63
Verbascoside	ph28	12.23	4.79	4.64	0.67	1.16	0.11	0.27	0.06	0.2	0.04
Luteolin-O-rutinoside isomer 2	ph29	0.5	0.11	1.04	0.12	0.27	0.03	0.13	0.05	0.05	0.02
Elenolic acid	ph30	866.6	170.96	1321	83.74	472.81	18.61	268.38	34.5	213.8	13.05
Luteolin-O-rutinoside isomer 3	ph31	0.13	0.06	0.26	0.03	TR	0	TR	-	TR	-
Nüzhenide	ph32	4.97	0.73	11.66	1.18	3.75	0.44	3.91	0.43	3.35	0.34
Luteolin-glucoside isomer 2	ph33	12.92	1.24	13.04	0.6	6.1	0.51	3.51	0.27	3.17	0.28
Caffeoyl-6-secologanoside	ph34	1.51	0.28	3.48	0.6	0.41	0.02	0.62	0.04	0.52	0.05
Luteolin-glucoside isomer 3	ph35	8.22	0.8	8.4	0.34	4.42	0.39	2.36	0.19	2.07	0.14
10-Hydroxy-DCMO aglycone ^F^	ph36	6.07	3.11	5.42	0.73	1.67	0.13	0.1	0.05	TR	-
Oleuroside	ph37	6.47	0.6	5.35	0.76	2.24	0.32	1.32	0.2	0.95	0.09
Comselogoside	ph38	5.55	0.89	9.06	1.69	2.4	0.37	1.7	0.1	1.31	0.18
Ligstroside derivative	ph39	0.24	0.17	0.75	0.16	0.09	0.09	TR	-	TR	-
Ligstroside aglicone	ph40	1.84	0.4	2.48	0.4	0.23	0.08	ND	-	ND	-
Luteolin	ph41	8.73	1.28	10.22	0.41	7.33	0.21	1.07	0.08	1.07	0.06
Apigenin	ph42	1.56	0.29	1.50	0.1	1.15	0.07	0.24	0.04	0.21	0.05
TOT ^G^	-	2007.34	416.65	2953.83	244.36	884.76	29.22	627.37	49.21	537.55	34.00

^A^ ND: not detected; TR: traces (compound detected under the limit of quantification); ^B^ Verbascoside residues lacking the rhamnose moiety; ^C^ Hydroxylated product of the dialdehydic form of decarboxymethyl elenolic acid; ^D^ Oleuropein aglycone decarboxymethyl dialdehyde form; ^E^ Ligstroside aglycone decarboxymethyl dialdehyde form; ^F^ 10-Hydroxy-decarboxymethyl oleuropein aglycone; ^G^ Total identified compounds.

**Table 6:** Sensory analysis results were reported as the number of assessors (out of 8–12 per session) for each attribute perceived by the panel involved in the hydroalcoholic extract (T0 and T2) evaluation.

Sample	Defects or Other Negative Attributes	Vanilla	Caramel	Red Fruits	Olive Fruits
T0	0	4	1	1	2
T2	0	2	2	3	3

## Data Availability

The data presented in this study are available on request from the corresponding author.
